# Harnessing and Enhancing Macrophage Phagocytosis for Cancer Therapy

**DOI:** 10.3389/fimmu.2021.635173

**Published:** 2021-03-10

**Authors:** Siqi Chen, Seigmund W. T. Lai, Christine E. Brown, Mingye Feng

**Affiliations:** ^1^Department of Immuno-Oncology, Beckman Research Institute, City of Hope Comprehensive Cancer Center, Duarte, CA, United States; ^2^Department of Hematology and Hematopoietic Cell Transplantation, City of Hope National Medical Center, Duarte, CA, United States

**Keywords:** macrophage, cancer immunotherapy, phagocytosis, antibody, chimeric antigen receptor (CAR), nanoparticle

## Abstract

Cancer immunotherapy has revolutionized the paradigm for the clinical management of cancer. While FDA-approved cancer immunotherapies thus far mainly exploit the adaptive immunity for therapeutic efficacy, there is a growing appreciation for the importance of innate immunity in tumor cell surveillance and eradication. The past decade has witnessed macrophages being thrust into the spotlight as critical effectors of an innate anti-tumor response. Promising evidence from preclinical and clinical studies have established targeting macrophage phagocytosis as an effective therapeutic strategy, either alone or in combination with other therapeutic moieties. Here, we review the recent translational advances in harnessing macrophage phagocytosis as a pivotal therapeutic effort in cancer treatment. In addition, this review emphasizes phagocytosis checkpoint blockade and the use of nanoparticles as effective strategies to potentiate macrophages for phagocytosis. We also highlight chimeric antigen receptor macrophages as a next-generation therapeutic modality linking the closely intertwined innate and adaptive immunity to induce efficacious anti-tumor immune responses.

## Introduction

Immunotherapy has had a long-standing history in the fight against cancer, with its early beginnings in the 19th century with Coley’s toxin (1). Up until now, about 40 biologics have been approved by the U.S. Food and Drug Administration (FDA) for patient use, with approximately 4,000 being actively investigated in clinical trials globally (2, 3). These modalities include immunomodulators (e.g., antibodies, antibody conjugates, cytokines, and immune agonists), vaccines, oncolytic viruses, and cell-based therapies (e.g., chimeric antigen receptor (CAR) T cell therapy) (2). As the exploration of novel immunotherapeutic targets and tumor-immunity interactions continue (4, 5), there is a growing interest in harnessing innate immunity to drive the development of effective immunotherapies for cancer.

The innate immune system, a major component of the body’s defense system, stands as the first line of defense against infectious pathogens and malignancies to maintain the body’s homeostasis (6). Innate immune cells are a diverse group consisting of effector cells such as natural killer (NK) cells and professional antigen-presenting cells like monocytes, macrophages, and dendritic cells (DCs). These cells rely on germline-encoded pattern recognition receptors (PRRs) and other cell-surface molecules to detect pathogen-associated molecular patterns (PAMPs) on invading microbes and tumor cells to orchestrate downstream responses (7). Furthermore, the innate immune system also cross-primes the adaptive immune system, during which antigen-presenting cells (APCs) process and present antigens to naive T and B cells, resulting in their activation (6). A precursor to this bridging of innate and adaptive immunity is APC antigen capture *via* phagocytosis, a multistep process closely regulated by the interaction of phagocytes and target cells (8). To evade detection and phagocytosis by the innate immune system, tumor cells exploit techniques normal cells use to label themselves as self-cells or counteract signals that can be detected by the innate immune system (9, 10). Thus, understanding the mechanism behind phagocytosis regulation could provide a new avenue for the development of next-generation therapeutic modalities, unleashing the power of innate immune system, especially macrophages, the most prominent tumor-infiltrating innate immune cell (11, 12).

Macrophages are highly efficient phagocytes capable of engulfing materials such as debris, dead cells, or pathogens (13). Tumor associated macrophages (TAMs) are a subset of macrophages that are abundant within the tumor microenvironment (14). They have demonstrated clinical significance in that they have been shown to contribute highly to tumor progression (15), resistance to therapies (16), and tumor metastases (17). M2 polarized TAMs are generally considered to have an anti-inflammatory phenotype and foster an immunosuppressive environment and produce anti-inflammatory cytokines and chemokines to benefit tumor growth (18, 19). M1 polarized TAMs have a pro-inflammatory phenotype and maintains an environment unfavorable for the tumor via pro-inflammatory cytokines to help hamper tumor growth. Both M1 and M2 polarized TAMs are capable of phagocytosing cancer cells (20), with the former being arguably superior (21). This function is largely mediated by the recognition of foreign materials mediated by the engagement of PRRs, scavenger receptors, and Fc receptors (22). For example, ligation of Fc gamma receptors (FcγRs) on macrophages with antibody Fc fragments initiates the process of antibody-dependent cellular phagocytosis (ADCP), an important mechanism linking innate and adaptive immunity.

In this review, we highlight recent advances made in enhancing macrophage by phagocytosis by targeting different stages of this process based on distinct principles. We first summarize the effects of therapeutic antibodies in inducing anti-cancer ADCP, followed by a discussion of strategies to promote ADCP-independent phagocytosis by macrophages, including nanoparticles and phagocytosis checkpoint blockade. Lastly, we will discuss recent breakthroughs in utilizing macrophages equipped with CARs for enhanced targeting and attacking of cancer cells. We aim to elucidate strategies ligating the closely intertwined innate and adaptive immune systems to elicit a superior anti-tumor response as a pivotal and modern effort to solve an age-old disease. Furthermore, we examine the implications this has on driving forward the field of immuno-oncology by challenging the status quo of standard cancer treatment and care.

## Antibody-Dependent Cellular Phagocytosis *Via* Therapeutic Antibodies

Monoclonal antibodies are an established paradigm for cancer treatment (23), achieving therapeutic efficacy not only by the antigen binding variable domains, but also the fragment crystallizable (Fc) domains. The Fc domain is bound by its corresponding immunoglobulin Fc receptor (FcR), a cell surface receptor family expressed by several hematopoietic cells, which includes IgG (FcγRI/CD64, FcγRII/CD32, and FcγRIII/CD16), IgE (FcϵRI), IgA (FcαRI/CD89), IgM (FcμR), and IgA/IgM (Fcα/μR) (24, 25). Within the human FcγR family, all but FcγRIIB are immunoreceptor tyrosine-based activation motif (ITAM) bearing activating FcRs that activate upon binding to IgGs *via* multimerization of intracellular ITAM domains (24, 26). FcγRIIB, on the other hand, is an immunoreceptor tyrosine-based inhibition motif (ITIM) bearing inhibitory FcR that dampens the activation of ITAM-bearing immune receptors (25), producing an immunosuppressive effect.

**Figure 1:**
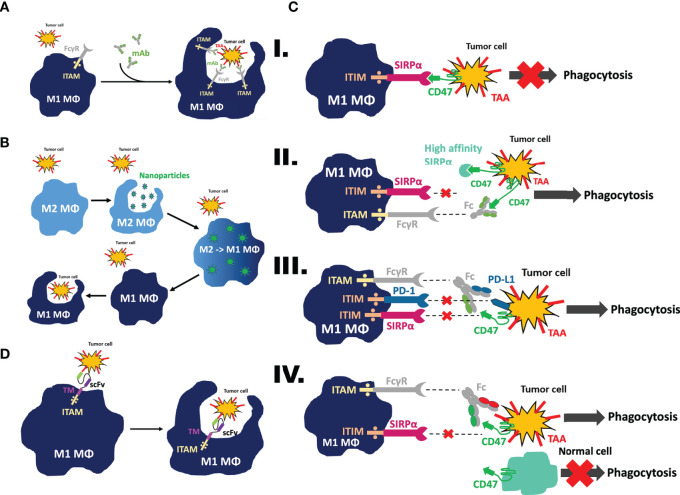
Mechanism of action to mobilize macrophages as effector cells against tumor cells. **(A)** Antibody-dependent cellular phagocytosis (ADCP). Following treatment with mAbs targeting tumor-associated antigens, Fc gamma receptors on macrophages will recognize the Fc domain of the antibody and trigger downstream activation of the immunoreceptor tyrosine-based activation motif (ITAM) to cause phagocytosis of the tumor cell. **(B)** Nanoparticle-mediated reeducation of M2 tumor associated macrophages (TAMs) into M1 TAMs. Nanoparticles will be recognized as foreign material and engulfed by M2 TAMs. Once this occurs, their contents will be released into the cytosol and trigger polarization of the macrophage away from the M2 pro-tumor phenotype toward the M1 anti-tumor phenotype. This process retrains the macrophage to perform phagocytosis on tumor cells. **(C)** CD47/SIRPα phagocytosis checkpoint blockade. I. Upon binding of CD47 on the tumor cell to SIRPα on the macrophage, an immunoreceptor tyrosine-based inhibition motif (ITIM) becomes activated, sparing the tumor cell from phagocytosis. II. Upon binding of the high affinity SIRPα fusion protein or anti-CD47 mAbs to CD47 on the tumor cells, or binding of anti-SIRPα mAbs to SIRPα on macrophages, the CD47/SIRPα axis is blocked and phagocytosis is restored. III. When a bispecific antibody is used, macrophage SIRPα and PD-1 ITIM activation is inhibited by a bispecific anti-CD47/PD-L1 antibody targeting the tumor cells, preventing the ligands from binding to its receptors; IV. In another scenario, the specificity of CD47 blockade is reinforced by dual-targeting of CD47 and tumor-associated antigen *via* a CD47/TAA bispecific antibody, therefore sparing normal tissue cells expressing CD47 but not TAA; the Fc region on the antibody is recognized by the FcγR on the macrophage, activating its immunoreceptor tyrosine-based activation motif (ITAM) and subsequently triggering phagocytosis of the tumor cell. **(D)** CAR-macrophages demonstrate enhanced phagocytic ability and tumor targeting specificity. When fitted with a CAR construct, macrophages are able to recognize tumor cells *via* their scFv region and trigger phagocytosis of the tumor cell. This occurs at a higher specificity and efficacy due to the CAR construct conferring increased tumor recognition capability to the macrophage.

ADCP is tumoricidal, as macrophages have been shown to phagocytose antibody-opsonized tumors across various preclinical models (**Figure 1A**). For example, ADCP is a critical and clinically relevant mechanism of action for daratumumab, a human monoclonal antibody targeting CD38, a glycoprotein found on immune cells, in multiple myeloma (27). Furthermore, ADCP is one of the cytotoxic mechanisms used by rituximab, ofatumumab, ocaratuzumab, and obinutuzumab, which are human monoclonal antibodies targeting CD20, a B cell surface protein, in chronic lymphocytic leukemia (28), as well as trastuzumab, an anti-HER2 monoclonal antibody that triggers phagocytic cytotoxicity of HER2^+^ cancer cells both *in vitro* and *in vivo* (29).

ADCP has also shown to be markedly dependent on FcγR. When transplanted with human breast tumor and B cell lymphoma xenografts, mice deficient in FcγRIIB exhibit a superior antibody-dependent cytotoxicity to tumor cells. That is, in the absence of the ITIM-bearing inhibitory Fc receptors, there is significantly more immune activation and cytotoxic effects exerted on target cells. This is in direct contrast to mice deficient in ITAM-bearing activating Fc receptors (FcγRI and FcγRIII), which results in impaired and overall inferior tumor growth inhibition upon treatment with the same antibody *in vivo* (30). This dependency on FcγR is further supported by *in vivo* experiments that demonstrate that macrophage depletion abrogates the ability for anti-CD20 antibodies to deplete B cells (31). Furthermore, colony-stimulating factor 1 (CSF-1) deficient mice with impaired macrophage development exhibit incomplete depletion of B cells upon treatment with anti-CD20 antibodies, while T or natural killer (NK) cells depletion had no impact on B cell clearance (31), emphasizing the importance of FcγR to propagate macrophage mediated ADCP.

Not only are macrophages professional phagocytes, they are antigen presenting cells as well. Thus, following ADCP of target cells, phagosomes containing tumor cells fuse with lysosomes for degradation, and tumor-derived antigen peptides are trafficked to major histocompatibility complexes (MHCs). This allows for the cross-presentation, activation, and priming of T cells (32). The breakdown of phagocytosed target cells within the phagosome often releases a significant amount of PAMPs. Nucleic acid sensors toll-like receptor 7 (TLR7), toll-like receptor 9 (TLR9), absent in melanoma 2 inflammasome (AIM2), and cyclic GMP-AMP synthase (cGAS) have been implicated in PAMP detection, subsequently activating inflammatory pathway cascades and causing the production and release of pro-inflammatory cytokines such as type I interferons, IL-1β, IL-6, and IL-12 (33–35).

Multiple lines of evidence have suggested that the inflammatory biological events in macrophages occur following ADCP. Interestingly, a recent study demonstrated that ADCP did not lead to an inflamed tumor microenvironment, but an immunosuppressive one for NK cells and CD8+ cytotoxic T cells* via *upregulation of PD-L1 and IDO expression by tumor-associated macrophages  (34). Admittedly, this phenomenon has thus far only been reported in breast cancers opsonized by Trastuzumab, and therefore warrants further investigation. Beyond this, it has also been reported that upon phagocytosis of Herceptin-opsonized HER2+ breast cancer tumor cells, AIM2 in tumor-associated macrophages is recruited and activated upon detection of tumoral DNA. This results in the cleavage of the IL-1β precursor and the release of bioactive IL-1β, stimulating the upregulation of PD-L1 and IDO in macrophages. Essentially, this phenomenon effectively inhibits antibody-dependent cellular cytotoxicity (ADCC) *via* NK cells and CD8+ cytotoxic T cells, but is rescued *via* PD-L1 and IDO blockade in addition to an anti-HER2 antibody (34).

Ultimately, the conclusion of these studies support the rationale of utilizing therapeutic strategies developed from a deeper understanding of macrophage immunology, either alone or in combination with standard of care therapeutic modalities, to create a more efficacious and favorable anti-tumor outcome. There is a dynamic interaction between macrophages and cancer cells during which macrophages detect and target cancer cells *via* the recognition of “eat me” signals which in many cases are exposed on cancer cells due to their intrinsic oncogenic stresses (36–38). On the other hand, tumor cells could utilize additional layers of “don’t eat me” signals, passing as self-cells, deceiving phagocytes to evade phagocytosis (36–38). Priming macrophages to enhance their ability in recognizing and targeting cancer cells, and/or blocking negative checkpoints or their ligands that transduce inhibitory signaling for phagocytosis have become attractive strategies for inducing the robust tumoricidal functions of macrophages (39). Moreover, the adoptive transfer of macrophages, especially *in vitro* tailored macrophages, to replenish tumor-infiltrating effector cells emerges as a novel avenue to boost tumor phagocytosis (40).

## Phagocytosis Checkpoint Blockade

Although the tumoricidal impact of ADCP is well-established, the complex relationship between tumors and macrophages has obscured the potential for harnessing tumor-associated macrophages as effector cells (18). Recent breakthroughs have highlighted phagocytosis checkpoint axes which can be targeted to induce the anti-cancer functions of macrophages, such as the CD47-SIRPα phagocytosis axis (41), PD1-PDL1 axis (10), MHC I–LILRB1 axis (42), and CD24–Siglec-10 axis (43). While antagonizing these phagocytosis checkpoints induces phagocytosis, the CD47-SIRPα axis is not only the best studied phagocytosis checkpoint, but also the only one with multiple therapeutic biologics entering clinical phase investigations with promising early-stage results.

CD47, also known as integrin-associated protein (IAP), is a ubiquitously expressed surface protein comprised of a long N-terminal extracellular domain, five transmembrane domains, and a short cytosolic tail (44). CD47 interacts with its binding partner, signal regulatory protein α (SIRPα), *via* its IgV-like domain with the N-terminal IgV-like domain of SIRPα (36). The CD47-SIRPα binding leads to the phosphorylation of two SIRPα intracellular ITIMs (45). Phosphorylated tyrosines in ITIMs recruit and activate SHP-1 and SHP-2, leading to the dephosphorylation of many proteins, including myosin IIA and paxillin (46). Activated SHP-1 and SHP-2, following CD47/SIRPα ligation at the phagocytosis synaptic interface, prevent integrin activation, as demonstrated by a recent study (47). As a result, cytoskeleton rearrangement is subsequently inhibited, and phagocytosis of target cells fails as well (**Figure 1C–I**). Antagonistic monoclonal antibodies are able to disrupt the binding of tumoral CD47 with SIRPα expressed on myeloid lineage cells, such as macrophages, dendritic cells, and neutrophils (41). Upon this blockade, the phagocytosis inhibition signal conferred by SIRPα is reversed, restoring phagocytic ability.

By exploiting this unique feature of CD47, it thus is an attractive therapeutic target to be used in clinical practice. As the most well-known anti-phagocytic signal, there have been and currently are a plethora of studies into using anti-CD47 targeting either as a standalone therapy, in combination with chemotherapy or to augment existing ADCP-inducing antibodies for example (48–50). Single agent therapies involving CD47-SIRPα axis blockade have been extensively discussed below. Studies have demonstrated that CD47 blockade synergizes with chemotherapy, radiotherapy (51), or ADCP, such as when in combination with Azacitidine (52), with anti-HER2 Trastuzumab (53), or with anti-CD20 Rituximab (54). It has also been reported that CD47 blockade not only mobilizes macrophages, but also activates dendritic cells, triggering phagocytosis of target cells and cross-presentation to the adaptive immune system (55, 56). Taken together, a wide array *in vitro* and *in vivo* studies have reinforced and supported the appealing therapeutic promise of exploiting CD47 blockade as a meaningful clinical practice to look toward.

Currently, the CD47-SIRPα axis is one of the most sought-after phagocytosis checkpoints in anti-tumor therapeutic development (**Figure 1C–II**) (57, 58). Multiple therapeutic biologicals designed to target CD47-SIRPα axis are now under extensive investigation in different developmental phases (36). A selection of such therapeutics targeting the CD47-SIRPα checkpoint currently in clinical trials has been summarized and compiled in **Table 1**. As of writing, the most advanced biologic is magrolimab, a humanized anti-CD47 monoclonal antibody (59), formally known as hu5F9-G4 (60–62). Magrolimab was the first-in-class therapeutic antibody, followed by many, to demonstrate that tumor-associated macrophages can be weaponized against tumor cells by blocking a phagocytosis checkpoint. Although magrolimab is generally well-tolerated in human clinical trials, anemia caused by on-target off-tumor binding of CD47 expressed on erythrocytes was a common treatment-related side effect (63). Given the wide therapeutic potential of anti-CD47 monoclonal antibody, one that effectively block the CD47 on tumor cells while sparing erythrocytes would be desirable (64). It is worth noting that the blockade of tumoral CD47 *via* a functional monoclonal antibody does not necessarily bring about hemagglutination, suggesting that anemia is not an unavoidable toxicity (65). To resolve this, a “priming dose” strategy was proposed and put to practice in clinics to mitigate the anemic side effects. 1 mg/kg of body weight magrolimab delivered intravenously eliminated aging red blood cells selectively while sparing ‘younger’ red blood cells, followed by a therapeutic dose of antibody treatment up to 45mg/kg one week later (63). In addition, tailored antibody screening and engineering was used when later anti-CD47 monoclonal antibodies were developed, such as Lemzoparlimab (TJC4), SRF231, and AO-176 (65–67), in which candidates causing hemagglutination or red blood cell (RBC) phagocytosis were pre-excluded alongside the characterization and ranking of their anti-tumor activity. As a result, these anti-CD47 antibodies maintain favorable phagocytosis of tumor cells facilitated by CD47 blocking, but exhibit a minimal to negligible amount of RBC related on-target off-tumor effect in preclinical *in vitro* and *in vivo* development (65–67). Currently, all three aforementioned anti-CD47 antibodies are in phase I clinical investigation. Interim analysis of lemzoparlimab in phase I clinical trial (NCT03934814) showed improved safety profile, escalated to 30 mg/kg without dose-limiting toxicity while achieved one confirmed PR in 30 mg/kg cohort (68).

**Table 1:** Current clinical trials involving CD47 blockade.

NCT Trial Identifier*	Drug Name	Target Disease(s)	Treatment Type	Current Phase	Status
NCT04435691	Magrolimab (Hu5F9-G4)	Recurrent acute myeloid leukemia	With azacitidine	Phase 1	Recruiting
		Refractory acute myeloid leukemia	With venetoclax	Phase 2	
NCT04541017		T-cell lymphoma	With mogamulizumab	Phase 1	Not yet recruiting
NCT02953509		Relapsed/Refractory B-cell Non-Hodgkin’s Lymphoma	With rituximab or with rituximab and chemotherapy	Phase 1/2	Recruiting
NCT03248479		Hematological Malignancies	Alone and with azacitidine	Phase 1	Recruiting
NCT04599634		Relapsed and Refractory Indolent B-cell Malignancies	With obinutuzumab and venetoclax	Phase 1	Not yet recruiting
NCT04435691		Acute myeloid leukemia	With azacitidine and venetoclax	Phase 1/2	Recruiting
NCT03869190		Advanced/Metastatic ureothelial carcinoma	With multiple different immunotherapies	Phase 1/2	Recruiting
NCT04313881		Myelodysplastic syndrome (MDS)	With azacitidine	Phase 3	Recruiting
NCT02663518	TTI-621	Hematological malignancies and solid tumors	Alone or with either rituximab or nivolumab	Phase 1	Recruiting
NCT03530683	TTI-622	Advanced relapsed/refractory lymphoma or myeloma	Alone or with either rituximab, PD-1 inhibitors, or proteasome inhibitors	Phase 1	Recruiting
NCT02367196	CC-90002	Advanced solid and hematological cancers	Alone and with rituximab	Phase 1	Active, not recruiting
NCT04485052	IBI-188	Acute myeloid leukemia	With azacitidine	Phase 1/2	Recruiting
NCT03763149		Advanced malignancies	Alone	Phase 1	Active, not recruiting
NCT04485065		High risk myelodysplastic syndrome (MDS)	With azacitidine	Phase 1	Not yet recruiting
NCT03717103		Advanced malignancies	Alone and with rituximab	Phase 1	Recruiting
NCT03834948	AO-176	Advanced solid tumors	Alone and with paclitaxel	Phase 1/2	Recruiting
NCT04445701		Relapsed/refractory multiple myeloma	Alone and with either dexamethasone or both dexamethasone and bortezomib	Phase 1/2	Recruiting
NCT04653142	BI 765063	Advanced solid tumors	Alone or with BI 765064	Phase 1	Recruiting
	BI 765064		Alone or with BI 765063		
NCT03990233	BI 765063	Advanced solid tumors	Alone or with BI 754091	Phase 1	Recruiting
NCT04417517	ALX-148	High risk myelodysplastic syndrome (MDS)	Alone and with azacitidine	Phase 1/2	Recruiting
NCT04675294		Advanced head/neck squamous cell carcinoma	Alone and with pembrolizumab	Phase 2	Recruiting
NCT04675333			Alone or with pembrolizumab or with pembrolizumab and chemotherapy	Phase 2	Recruiting
NCT03013218		Advanced solid tumors and lymphoma	Alone or with either pembrolizumab, trastuzumab, rituximab, pembrolizumab and 5FU and platinum, or trastuzumab and ramucirumab and paclitaxel	Phase 1	Recruiting
NCT04097769	HX009	Advanced malignant tumors	Alone	Phase 1	Recruiting
NCT04202003	TJ011133	Relapsed/refractory AML or MDS	Alone	Phase 1/2	Recruiting
NCT03934814		Relapsed/refractory advanced solid tumors and lymphoma	Alone or with either pembrolizumab or rituximab	Phase 1	Recruiting

*Information adapted from clinicaltrials.gov is current as of 10 January 2021.

Beyond employing antibodies to block CD47, another effective and popular approach is by using recombinant proteins (**Figure 1C–II**). These CD47 antagonist recombinant proteins are designed by first fusing an engineered fragment derived from the N-terminal V-set Ig domain of SIRPα’s extracellular domain with a Fc region of an antibody. As of writing, the most advanced fusion products developed are TTI-621 (69), TTI-622 (70), and ALX-148, previously known as CV1 (60, 61). TTI-621 and TTI-622 are derived from the natural human SIRPα allelic variant V2, harboring a 12 amino-acid mutation to the allelic variant V1 commonly referred to the SIRPα. TTI-621 and TTI-622 preferably bind with tumoral CD47 with high affinity, but minimally to CD47 expressed on erythrocytes. Taking advantage of this selectivity, the two SIRPα-Fc fusion proteins achieved a biased CD47 binding selectivity, thereby facilitating phagocytosis of tumor cells while avoiding hemagglutination in patients (62, 70). It is also worth noting that TTI-621 and TTI-622 vary only in their Fc isotype. TTI-621 is fused to a human IgG1 Fc domain, whereas TTI-622 to a human IgG4 Fc domain, therefore, differential activating signals through Fcγ receptors in addition to CD47 blockade can be generated from these two fusion proteins. However, because the overall safety profile of the effector functions of Fc fragment in conjugation to CD47 blockade was not fully determined at the time, both therapeutic biologics were developed preclinically. Both TTI-621 and TTI-622 are currently undergoing clinical trial investigation against hematologic malignancies (NCT03530683, NCT02663518)

On the other hand, ALX-148 is able to saturate CD47 receptors and easily outcompete endogenous wildtype SIRPα due to its extraordinarily high binding affinity. However, because ALX-148 is engineered based on the natural SIRPα allelic variant V1, it does not differentiate between CD47 expressed on tumor cells and RBCs  , therefore anemia is still observed in clincial trials (71). The approach of using fusion proteins is largely similar to using anti-CD47 monoclonal antibodies with regard to their mechanism of action. Both arms result in a blockade of the CD47-SIRPα axis, but the fusion protein strategy may have certain advantages over the monoclonal antibody approach, such as superior binding affinity and biodistribution. Taking ALX-148 for example, generated *via* yeast display and directed evolution, its binding affinity has been enhanced to 11 pM (72), whereas hu5F9-G4, generated from hybridomas, binds with CD47 at an affinity of 8 nM, 2 magnitudes lower than ALX-148 (73). Fusion proteins weigh roughly half the molecular weight of a monoclonal antibody, therefore it is putative to be easier for fusion proteins to penetrate into solid tumors, as it has been shown that smaller molecules can infiltrate solid tumors easier through leaky capillary vessels *via* simple diffusion (74, 75). However, no evidence thus far has supported an improved biodistribution profile for ALX-148 to the best of our knowledge

However, therapeutic strategies targeting the CD47-SIRPα axis do not stop at just blocking CD47. Several functional anti-SIRPα monoclonal antibodies have been reported, serving as an antagonist for the CD47-SIRPα signal (59, 76–78). For example, the anti-SIRPα mouse antibody KWAR23 demonstrated enhanced neutrophil and macrophage anti-tumor abilities in human SIRPα knocked-in mice. When synergized with rituximab, the growth of human Burkitt’s lymphoma xenograft models were profoundly inhibited (76). Humanized anti-SIRPα antibody 1H9 is the latest reported therapeutic antibody, showing broad-spectrum binding to several SIRPα variants without cross-reacting with other SIRP family members. When synergized with other therapeutic antibodies in various preclinical *in vivo* models, 1H9 demonstrates a good safety profile in non-human primates with less antigen sink compared to anti-CD47 antibody 5F9 (78). Unlike ubiquitously expressed CD47, SIRPα expression is largely restricted to cells of myeloid lineages. Because of this, specifically targeting SIRPα, as opposed to CD47, could bring about differentiated safety and efficacy of therapeutic models, therefore allowing for an improved therapeutic index.

CD47-SIRPα “don’t eat me” axis blockade has been proved so far to be a great success in mobilizing tumor-associated macrophages for tumor cell eradication (57). Various improvements for therapeutic monoclonal antibodies were implemented in pursuit of better therapeutic profiles. However, monoclonal antibodies are sometimes handicapped by their mechanism of action, in that their biodistribution could lead to an unfavorable pharmacokinetic profile, bottlenecking clinical efficacy. Indeed, though CD47 blockade therapies have achieved early therapeutic efficacy in acute myeloid leukemia (AML) and myelodysplastic syndrome (MDS) patients in clinical trials (79, 80), this strategy has so far struggled in coping with solid tumors. Furthermore, it is possible that monotherapy acting on a single target is simply insufficient to overcoming the heterogeneous tumor microenvironment (81).

## Systematic Engagement of Macrophages by Bispecific Antibody

Given the increasing awareness and interest in tumor heterogeneity, bispecific antibodies have become a promising strategy to combat cancer and other diseases. In contrast to targeting T cells or NK cells through a bispecific antibody (82, 83), this principle has remained understudied in macrophages. To the best of our knowledge, the concept of approaching macrophages as tumoricidal effectors by bispecific antibody was first proposed in 2015 (13). As of writing, only a handful of bispecific antibodies designed to exploit macrophages have been reported, with even less validated in clinical trials.

Unlike T cell engagers, which require a specific antibody variable domain arm for engagement (84), macrophages can recognize and bind to the Fc fragment of an antibody *via* FcγR recognition, followed by the phagocytosis of opsonized target cells (13). This unique feature licenses bispecific antibodies adopting IgG-like formats to recruit macrophages without variable domain engagement. Therefore, variable domains of bispecific antibodies in this category can be exploited to target tumor-associated antigens and “don’t eat me” signals on target cells simultaneously, harnessing the power of ADCP and phagocytosis checkpoint blockade at the same time to enhance phagocytosis of tumor cells.

The role of CD47 in immune evasion as well as therapeutic potential of CD47 blockade was first described in an AML model (9, 41). Following this study, HMBD004, an anti-CD47/CD33 bispecific antibody, was developed based on a humanized anti-CD47 antibody and anti-CD33 gemtuzumab, adapting a 1 + 1 IgG format (85). This bispecific antibody maintained CD47-SIRPα axis blockade as well as phagocytosis induction with negligible hemagglutination of erythrocytes *in vitro*. Furthermore, HMBD004 treatment of AML xenograft mice models resulted in a significant decrease in tumor burden and increased progression-free survival (85). Another notable example of a bispecific engager was noted in NI-1701, a CD47/CD19 bispecific antibody constructed with the IgG1 isotype to elicit an ADCP response. This bispecific antibody demonstrated potent *in vitro* and *in vivo* activity across a plethora of B cell malignancy models, which rely on the co-engagement of CD47 and CD19 on B cells simultaneously to induce potent ADCP of target cells (86, 87).

With the success of the anti-CD47 antibody Magrolimab (hu5F9-G4) in combination with Rituximab in clinical trials treating B-cell non-Hodgkin’s lymphoma (54, 88), a logical bispecific antibody design strategy is to combine the anti-CD47 arm with another arm targeting a B-cell specific antigen, such as CD20 (89, 90) or CD19 (86, 87, 91) (**Figure 1C–IV**). Two bispecific antibodies were recently reported adapting this strategy: one fused an anti-CD20 variable domains at the N-terminal of the variable domains of an anti-CD47 antibody (89); the other fused with the N-terminal V-set Ig domain (residues 1-118) of SIRPα to the N-terminal of the heavy-chain variable domain of an anti-CD20 antibody (90). Both bispecific antibodies exhibited improved tumor cell targeting while avoiding any on-target off-tumor effect. Furthermore, both bispecific antibodies resulted in superior *in-vivo* efficacy in Raji xenograft, conferring improved tumor growth inhibition and prolonged survival in comparison to parental antibody monotherapy and combination therapy. Furthermore, a recent study in 2017 engineered a novel bispecific antibody known as RTX-CD47 with the ability to target both CD47 signaling as well as CD20-positive cells. To accomplish this, the CD20-targeting scFV antibody fragment from rituximab was fused to a CD47-blocking scFv. *In vitro* examinations conducted in various CD20 expressing cell lines demonstrated superior macrophage-mediated phagocytosis, particularly when used in synergy with therapeutic antibodies such as cetuximab, daratumumab, alemtuzumb, rituximab, or obinutuzumab (92).

In addition to targeting tumor antigens, it is also of interest to target multiple immune checkpoints, such as CD47 and PD-L1. PD-L1 is generally overexpressed on tumor lesions, and thus targeting it could help to improve the retention of CD47/PD-L1 bispecific antibody in tumor tissue (**Figure 1C–III**). Not only is PD-L1 a canonical T cell checkpoint (93), it has also been recently identified as a macrophage “don’t eat me” signal. Therefore, its blockade has been shown to reinvigorate the anti-tumor function of TAMs (10). Because of this, a CD47/PD-L1 bispecific antibody could unleash more potent macrophage phagocytosis ability. In a recent proof-of-principle study (94, 95), the pre-clinical anti-tumor efficacy of such a bispecific antibody was tested in multiple synergistic mouse models. In comparison to anti-CD47 or anti-PDL1 monotherapy or anti-CD47 + anti-PDL1 combinational therapy, simultaneously targeting both CD47 and PDL1 on tumor cells with a CD47/PDL1 bispecific antibody delivered the best tumor growth inhibition and prolonged recipient survival (94, 95). Mechanistically, the systemic delivery of the dual-targeting agent significantly increased DNA sensing, dendritic cell cross-presentation, and an anti-tumor T cell response (94, 95).

Another notable example of a bispecific antibody was constructed by researchers at Stanford University to target CD70 and SIRPα by fusing the variable domain of Vorsetuzumab, an antibody targeting CD70, a protein expressed on activated lymphocytes, to the N-terminal of corresponding variable domain of KWAR23, an anti-SIRPα antibody. The CD70/SIRPα bispecific antibody was able to target CD70-expressing cells including NHL and renal cell carcinoma and facilitate the engagement of macrophages. When compared to Vorsetuzumab + KWAR23 treatment, the CD70/SIRPα bispecific antibody demonstrated an enhanced *in-vitro* phagocytosis of target tumor cells, but there was no apparent difference in *in vivo* efficacy observed (76). This demonstrates that the bispecific antibody is able to function on a par with a combinatory treatment of both arms of the antibody separately (Vorsetuzumab + KWAR23). Given that this only requires the administration of a single biologic, it eliminates the possible confounding variable associated with balancing the administration and controlling for interactions between two separate biologics. Pharmacokinetically speaking, this would have implications warranting further study into the dosing and safety profile of such an antibody.

## Immuno-Quiescent Bispecific Antibody

Phagocytosis is crucial for maintaining homeostasis. A bispecific antibody to bring together macrophages and phagocytosis targets would be of particular interest in cases where tissue homeostasis restoration is key, without inciting an inflammatory response. Such scenarios include but are not limited to degenerative central nervous system diseases, autoimmune diseases, and pandemics like severe acute respiratory syndrome coronavirus 2 (SARS-CoV-2).

Recently, a team at Genentech performed a proof-of-principle validation of a MerTK-agonist bispecific antibody (96). MerTK is a part of the Tyro3-Axl-MerTK family of receptor tyrosine kinases and are indispensable for maintaining tissue homeostasis. Guided phagocytosis through agonism of MerTK was shown to be inflammation-quiescent, in sharp contrast to the inflammatory nature of FcγRs-mediated phagocytosis, namely ADCP (96–98). By exploiting LALAPG mutations in Fc fragments (L234A, L235A, and P329G) to completely abolish Fc mediated effector function (99, 100), the CD20/MerTK bispecific antibody (CD20/18G7-LALAPG) has been shown to induce antigen-specific target cell phagocytosis through activation of MerTK in human macrophages with negligible production of pro-inflammatory cytokines (96). This bispecific antibody model was further engrafted with an anti-Aβ amyloid plaque arm. The Aβ/MerTK bispecific antibody, 3D6/20F5-LALAPG, elicited improved Aβ aggregate clearance by microglial cells, but not the production of inflammatory cytokines (96).

## Facilitating Phagocytosis Using Nanoparticles

Seeing as ADCP is in some cases insufficient to mount an effective anti-tumor response, a potential method of invigorating the phagocytic response is using nanoparticles. The use of different nanoparticles, such as silica, carbon, iron oxide, or gold to drive macrophage polarization states has been extensively studied in literature (101). When TAMs recognize the nanoparticles as foreign, they will engulf them *via* phagocytosis, releasing the contents of the nanoparticle within the TAMs (102). Therefore, nanoparticles can be packed with drugs or contents designed to induce macrophage polarization toward a more phagocytic phenotype to reprogram them with an affinity for phagocytosis, thus making them an attractive vehicle for therapy delivery (**Figure 1B**) (101, 103, 104).

Nanoparticles are an appealing therapeutic vehicle largely due to their physical characteristics and small size (105, 106). Their appeal is further reinforced by the phenomenon of Enhanced Permeability and Retention (EPR) effect, which in tumor tissue helps to promote the accumulation and persistence of nanomedicines at the site of the tumor (107, 108). The leaky nature of the tumor vasculature allows for nanomedicines to penetrate into tumors, where the minimal lymphatic drainage and filtration facilitates their accumulation and persistence (109, 110). As of writing, there are over 200 clinical trials currently underway globally investigating the use of different nanoparticles against different cancers, primarily solid tumors (111). Patient use of several nanoparticles has already been validated and approved by the FDA (112).

However, the prospect of nanoparticles delivering drugs to specific subsets of macrophage phenotypes has been relatively understudied (113). A recently published study was among the first to engineer nanoparticles capable of preferentially targeting M2 TAMs through the delivery of nanoparticles carrying M1-polarizing transcription factors and mRNAs for interferon regulatory factor 5 (IRF5) as well as IKKB, its activating kinase (114). The delivery of both IRF5 and IKKB were able to force M2 TAMs to polarize to a pro-inflammatory state and convert to a M1 cytotoxic phenotype (114). A unique quality of this method is its method of administration *via* injection that does not trigger an immune reaction or lead to systemic toxicity in the recipient. Another innovative aspect about this study is how the authors generated the targeted mRNA delivery system to target mannose receptors on M2 macrophages. When tested *in vivo¸* treatment with IRF5/IKKB NPs led to a significant regression of ovarian cancer and was able to be cleared and prolonged the lifespan of the mice (114).

This finding was supported by a later study in which researchers loaded IMD-0354, a TAM repolarization agent, into mannose-modified cationic lipid-based nanoparticles (M-IMD-CLN), and sorafenib, a kinase inhibitor used to treat cancer, into cationic lipid-based nanoparticles (SF-CLN) (115). Mannose is selectively taken up by M2 TAMs, which are characterized as highly expressing mannose receptors. Therefore, the mannose present on the nanoparticles become the ligand for M2 mannose receptors and thus enhance its active uptake by M2 TAMs, as demonstrated by the *in vivo* experiments exhibited a superior biodistribution profile and localization to the site of the tumor, eliciting TAM potent anti-tumor properties in Hepa1-6 tumor bearing mice (115). In addition to this, when M-IMD-CLN and SF-CLN are used together in junction, there appears to be a synergistic augmented anti-tumor efficacy and TAM re-polarization compared to mice treated with SF-CLN alone (115).

Later, Chen et al. further reinforced the potential for using mannose as a ligand for targeted delivery of nanoparticles to M2 TAMs by conjugating bivalent ligands for mannose receptors onto nanoparticles, effectively driving their uptake by M2 TAMs (113). This was accomplished by differentiating rat peritoneal macrophages into M1 *via* IFN-y and M2 *via* IL-4/IL-13 treatment. Markers distinguishing between resting, M1, and M2 macrophages were assessed to confirm their phenotypes. These groups of macrophages were then treated with the bivalent mannose nanoparticles and their uptake was quantified. These nanoparticles were found to have a significantly higher level of uptake in M2 macrophages, especially when compared to M1 or resting macrophages (113).

It is important to note that nanoparticles are not limited to containing only a single drug or compound to be delivered (116). Multivalent nanoparticles have been studied extensively for their potential to carry multiple drugs or compounds, thus hitting multiple targets (117, 118). For example, albumin-based nanoparticles expressing transferrin receptor binding peptide T12 and mannose were capable of not only polarizing M2 TAMs to become M1, but also remodeling the tumor microenvironment to allow for an enhanced anti-tumor response (119). Gliomas highly express transferrin receptor and albumin-binding receptor SPARC, while M2 TAMs also highly express SPARC and mannose, therefore facilitating the dual-targeting role of these nanoparticles for both gliomas and M2 TAMs. When these nanoparticles are loaded with both disulfiram/copper complex, a treatment for glioma, and regorafenib, a kinase inhibitor that repolarizes TAMs to M1, glioma proliferation was inhibited and M2 TAMs were re-programmed toward a M1 phenotype (119, 120). Thus, these studies and others provide a proof-of-principle that nanomedicines can be designed to preferentially target M2 TAMs. Future investigations could study arming these nanoparticles with drug payload with a final destination of M2 TAMs to either inactivate or reprogram them. Therefore, when considering the use of nanoparticles to facilitate and enhance macrophage-mediated phagocytosis, the following main approaches are of interest: 1) inactivating or eliminating M2 TAMs, and 2) reprogramming M2 TAMs to acquire a M1 pro-inflammatory phenotype to enhance phagocytosis.

Furthermore, a multi-functional protein calreticulin has been demonstrated as an important pro-phagocytic “eat me” signals for apoptotic cells and many types of cancer cells. In cancer cells with apoptosis induced by chemotherapeutic agents such as anthracyclines and oxliplatin, calreticulin trafficks to the cell surface, dictating a process of immunogenic cell death (ICD) (121). ICD is characterized as an immunostimulatory process that triggers an adaptive immune response against certain epitopes of antigens, including tumor neo-epitopes (122). In addition, macrophages have been shown to be an important resource for calreticulin (123–127). Macrophages present calreticulin on their cell surface or secrete it to the extracellular media to directly label unwanted cells such as aging or malignant cells for subsequent phagocytosis. Interaction between calreticulin and asialoglycans on unwanted cells enables their recognition by macrophages and initiation of phagocytosis. A recent study demonstrated that macrophage-mediated anti-tumor immunity was found to be enhanced upon intratumoral injection of nanoparticles containing calreticulin (128). Yuan et al. designed dual-function colloidal nanoparticles capable of 1) targeting HER2, a receptor commonly expressed on cancers, and 2) promoting anti-tumor phagocytosis *via* calreticulin (129). In this case, calreticulin promotes phagocytosis of target cells by enhancing APC recognition of target cells. In junction with the HER2 targeting, engagement of this dual-function nanoparticle with APCs augments the anti-tumor response by activating both the innate and adaptive immune system with one particle (129). Effectively, these allow for a synergistic effect achieved through a combinatory therapy being delivered within a single particle that may be superior to either therapy alone. Particularly, this allows for the selective targeting of M2 TAMs to either inactivate them to remove their immunosuppression or reprogram them into a M1 phenotype to elicit anti-tumor immunity.

However, a significant barrier for using nanoparticles is the fact that despite being able extravasate and persist due to EPR, only a very small percentage (less than 1%) of injected nanoparticles are actually able to traffic and be delivered to their intended destination (130, 131). As such, this remains a critical handicap to the efficacy of using nanoparticle-based treatments in patients. It remains a challenge for the nanoparticles to be homed and actively directed to the site of the tumor, rather than rely on passive targeting (132).

A study conducted in 2019 highlighted the potential for using exosomes derived from effector CAR-T cells as they retain their anti-tumor capabilities with minimal toxicity or cytokine storm (133). This method could thus be used to mitigate the problem of poor trafficking and localization of nanoparticles to their destination. That is, the “CAR exosomes” retain the CAR expressed on their host cells, allowing these nanoparticles to maintain their parental cells’ unique targeting feature (133). Conversely, rather than taking exosomes from CAR-T cells, another recent study demonstrated this phenomenon in nanoparticles coated with the cell membrane of CAR-T cells, therefore conferring them target antigen specificity similar to CAR-T cells (134). In this study, the CAR-T cell membrane coated nanoparticles demonstrated a superior tumor targeting ability compared to uncoated nanoparticles (134). Both of these findings open the door to future investigation into how to maximize nanoparticle infiltration, persistence, and targeting to overcome these barriers in order to enhance their therapeutic efficacy.

## Chimeric Antigen Receptor Engineered Macrophages

Despite the increasing interest in CAR-T cell therapies, there has been a struggle in reproducing its therapeutic efficacy observed in hematological malignancies in solid tumors (135–137). This is heavily related to the biology of how T cells fight tumor cells, as they need to be presented with antigens, primed, trafficked to the tumor, infiltrate tumor tissue, and recognize and kill malignant cells (81, 138). However, due to the heterogeneous nature of tumor tissue and the complex immunoinhibitory tumor microenvironment, not all of these critical events can occur successfully (74, 136, 139–142).

To address these roadblocks, CAR T cell therapy has recently turned to macrophages. Macrophages are known to be abundant and actively recruited to various types of solid tumor tissue (17, 143, 144). Recruited by macrophage chemo-attractants (i.e, CCL2, MCP1, CSF-1), tumor-associated macrophages represent up to 50% of tumor-infiltrating cells, as seen in melanoma, renal cancer, and colonic carcinoma (145, 146), suggesting they may be able to efficiently infiltrate into solid tumors upon adoptive transfer. Not only this, but macrophages have a unique quality in that they have a high degree of versatility and plasticity (147), and are thus able to adapt and change in response to external stimuli or the environment (148). The abundance and plasticity of tumor associated macrophages corroborate the idea of fitting macrophages with CAR constructs, therefore conferring target antigen specificity, and promoting engineered macrophages to efficaciously target solid tumors (**Figure 1D**). However, it is worth noting here that although their plasticity may provide therapeutic versatility, it may also act as a potential pitfall. In particular, although plasticity can be a desired trait, it is only so if they skewed toward a M1 pro-inflammatory phenotype that will target the tumor. Furthermore, macrophage activation has been associated with the development of macrophage activation syndrome (MAS), characterized by a surge in pro-inflammatory cytokines, resulting in a cytokine storm (149, 150). Ultimately, this warrants further investigation to see how to prevent or mitigate these phenotypes *in vivo* to minimize unwanted side effects and enhance treatment efficacy and most importantly, patient safety.

The construction of CARs for macrophages requires similar components to that of T cells: a target binding extracellular domain adapted from an antibody, followed by a hinge sequence, a transmembrane domain connecting the extracellular domain and intracellular signaling domain (136). It is worth to keep in mind the differences of signal pathway required for T cell activation and macrophage phagocytosis when designing a CAR for macrophages. The intracellular domains of CAR-macrophages reported so far have varied from that of CAR-T cells. CD3ζ is a common intracellular domain being studied in both, while CD147, FcγR, and Megf10 have been studied more exclusively in CAR-macrophages (CAR-Ms) only, for example (40, 151, 152). Phagocytosis-oriented CAR constructs are capable of rewiring macrophages and jumpstarting phagocytosis. Theoretically, CAR expression by macrophages would not only help the effectors to target cells, but also to circumvent the need of an “eat me” signal, to initiate phagocytosis signaling.

## Current Studies of CAR-Macrophages

Up until now, the study of utilizing CAR-Ms, has been relatively limited. A recent study by Morrissey and colleagues was among the first to provide initial evidence that CAR-engineered macrophages can promote phagocytosis. By utilizing an extracellular antibody variable fragment (ScFv) targeting CD19 as well as the CD8 transmembrane domain present in a traditional CD19 CAR-T construct, researchers were able to generate chimeric antigen receptors for phagocytosis (CAR-Ps) on murine macrophage cell line J774A.1 that successfully drove the phagocytosis of antigen-coated decoy particles in an *ex vivo* setting (151). In this study, the authors were able to test phagocytosis specificity guided only by the antigen recognition feature of the ScFv domain of the CAR construct. The authors were also able to compare the capacity of driving phagocytosis by various intracellular domains in a relatively simplified *ex vivo* assay (151).

The potential for engineering macrophages with CAR constructs was further reinforced by another study that elected to generate murine CAR-Ms specifically targeting the solid tumor antigen HER2 that utilized the intracellular signaling domain of CD147 to drive expression of matrix metalloproteinases (MMPs) in a HER2-dependent manner. To accomplish this, the authors first forced HER2 expression on 4T1 tumor cells and transduced murine macrophage cell line Raw264.7 with the HER2-147-CAR (152). From this, they were able to induce matrix MMP3 and MMP13 expression specifically upon ligation of the CAR and its antigen to remodel tumor microenvironment, increase T cell infiltration, and reduce tumor growth *in vivo* (152).

A study published earlier this year was the first to successfully generate human CAR-Ms with demonstrated functionality in both *in vitro* and *in vivo* models (40). CAR-expressing human macrophages were generated with a chimeric Ad5/F35 adenovirus (40, 153). Using CAR constructs with the CD3-ζ intracellular signaling domain and ScFvs targeting CD19, HER2, and mesothelin, the authors showed that antigen specific tumor cell killing was triggered by the CAR-transduced human macrophage line THP-1. Importantly, the authors establish that primary human macrophages can also be engineered to express a CAR to promote phagocytosis, and showed that HER2-CAR Ms exhibited dramatic *in vivo* therapeutic efficacy in various preclinical xenograft models. Interesting, transduction using Ad5/F35 adenovirus polarized human macrophages to a pro-inflammatory M1 state independent of CAR expression. Furthermore, CAR-Ms recognition of tumor cells shifted the tumor microenvironment from an anti-inflammatory one to a pro-inflammatory one, activating several interferon genes and inflammatory pathways along the way (40). Taken together, these findings suggest that CAR-Ms can both induce tumor phagocytosis, as well as reshape the tumor microenvironment to promote anti-tumor T cell responses. A Phase 1 preclinical trial (NCT04660929) investigating the safety and early-stage efficacy of HER-2 CAR-Ms is underway.

Collectively, these studies represent the first proof-of-principle validation of using CAR-M as an immunotherapeutic. CAR-Ms not only function *via* direct target cell phagocytosis, but also through vaccinal antigen cross-presentation and T cell co-stimulation (40). Macrophages have been shown to reside and persist within tissues, and certain tissue-resident macrophages are able to survive quiescently for relatively long period of time until challenged (154–156). This very feature makes CAR-Ms the ideal local sentinels for metastatic tumor cells, thus preventing tumor relapse at early metastasis stage. It is worth noting that the plasticity of macrophages endows itself the potential for application beyond tumors where inflammation is dispensable. With the proper design of CAR constructs, macrophages can be converted into immunoquiescent CAR-Ms while preserving their capacity to phagocytose, thus addressing other unmet medical challenges such as pathogen infection, autoimmune diseases, and degenerative neural diseases. Given the vast potential, CAR-Ms can transform the current landscape of cancer care and become a next-generation therapy for cancers and beyond.

## Conclusion

The mechanisms of macrophage phagocytosis and subsequent adaptive immune cross-priming have been increasingly appreciated and studied as a crucial effector function complementing adaptive anti-tumor immune responses. Antibody-dependent cellular phagocytosis has long been extensively studied and applied in clinical settings for at least 20 years. For example, ritxuimab was the first-in class monoclonal antibody approved by the FDA in 1997. Since then, this list has expanded to include a plethora of novel antibodies of varying designs that are now approved or undergoing clinical trial evaluation. Although this exciting field of study first began with the initiation of ADCP *via* therapeutic antibodies, it has now gone on to expand into exploiting phagocytosis checkpoints and beyond to enhance therapeutic efficacy, leading to novel fields of study including: 1) using nanoparticles to engineer macrophages with enhanced phagocytic ability, 2) strategies to block self-protective signals on cancer cells, and 3) fitting CAR constructs on macrophages to confer target antigen specificity. These exciting breakthroughs have marked an important step forward as macrophage-mediated phagocytosis of cancer has begun to enter the spotlight as a promising novel cell-based therapy.

The CD47–SIRPα axis was the first phagocytosis checkpoint discovered in cancer (41), and new phagocytosis checkpoints have since been identified, such as PD1-PDL1 (10), MHC I–LILRB1 (42), and CD24–Siglec-10 (43). Phagocytosis checkpoint blockade therapies have thus far resulted in promising human trial data, such as CD47 blocking therapeutics Magrolimab in NHL and AML, or TTI-622 in lymphoma (52, 70). By the nature of different yet complementary immunological pathways mobilized upon phagocytosis checkpoint blockade, macrophage phagocytosis proves itself as a novel and promising therapeutic tool on par with many approved treatments, such as PD-1 checkpoint blockade (157). Given the heterogeneity of solid tumor cells, and the suppressive and often immune exclusive tumor microenvironment, phagocytosis checkpoint blockade appears to be a promising starting place for effective anti-tumor innate immune responses. Whether phagocytosis checkpoint blockades can serve as a vehicle, either in the form of monoclonal antibody or bispecific antibody, to conquer solid tumors, especially hard-to-treat metastatic tumors, warrants further intense investigation.

There are also several clinical trials underway to evaluate therapies specifically targeting TAMs *via* nanoparticles. These trials and other original research studies have yielded promising data that can hopefully be applied to patients soon (158, 159). However, in order for these therapies to be optimally translated into patient use, there is much work that still needs to be done to prove TAM targeting *via* nanomedicine is feasible. Thus far, promising preliminary results suggests there is vast potential in which these nanoparticles can be applied, even beyond just targeting TAMs (116). Despite this, more proof-of-principle and validation studies need to be conducted before its use can be optimized and integrated into standard practice for cancer patient care. For example, a critical question that remains to be answered is how to optimize and maximize the number of nanoparticles that will actually traffic and localize to the tumor. As demonstrated in countless studies, despite being bolstered by EPR to enhance nanoparticle accumulation *via* leaky vasculature, only around 1% of the nanoparticles will actually make it to the site of the tumor (130, 131). This is an important first roadblock that critically needs to be overcome before expanding into the plethora of possibilities surrounding nanomedicines. Furthermore, another question of interest is how to facilitate the persistence of these nanoparticles *in vivo* such that they are not rapidly eliminated by the immune system. Even if the particles are able to overcome the low trafficking and tumor localization rate, another factor to consider is the body’s immune system perceiving them as foreign threats and eliminating them before they are able to exert their therapeutic effect. To address this, it would be interesting to apply a principle studied in Deuse et al. in generating hypoimmunogenic nanoparticles that evade immune detection and elimination to allow them to persist and ensure drug delivery through the suppression of MHC I and MHC II while overexpressing anti-phagocytic signal CD47 (160). Seeing as these are major handicaps dampening the full potential and efficacy of nanomedicine-based approaches to elicit potent M1 TAM activity, there ultimately remains much work to be done to study this exciting and novel form of immunotherapy.

Last but not least, the prospect of CAR-M based immunotherapy was propelled forward by previous findings by Morrissey et al. and Zhang et al. (151, 152). The Klichinsky et al. study marked a critical breakthrough in reinforcing the efficacy of macrophage-based therapeutics against cancer (40). By fitting macrophages with a CAR construct, they leveraged the potency of macrophage-mediated phagocytosis in conjunction with target antigen specificity to elicit potent anti-cancer effects that appear to be on par with their CAR-T counterparts. Not only were they able to successfully overcome the difficulties with transducing primary macrophages, they were also able to exploit their plasticity to drive them toward a M1 phenotype while simultaneously remodeling the tumor microenvironment to be pro-inflammatory. These exciting findings are now beginning to be put to the test clinically, beginning with a CAR-M against HER2. Though the use of CAR-Ms holds great promise as an innovative immuno-oncology therapeutic avenue, there is still a need for the development of a pipeline investigating its efficacy and safety profile. As many studies have demonstrated in their CAR-T counterparts, cytokine release syndrome remains one of the main obstacles to overcome. Cytokine release syndrome is largely mediated by the activation of macrophages, triggering an overwhelming sudden release of pro-inflammatory cytokines (161). As such, investigations into the safety profile of CAR-Ms would be particularly interesting to delve into. In addition to this, another question of interest is how to design CAR constructs and strike a balance between maximizing phagocytosis signals and enhancing the CAR-M’s specificity while minimizing unwanted toxicity. Collectively, this could be an emerging direction of study in this promising field.

Ultimately, recent breakthroughs in the exciting field of harnessing macrophage-mediated phagocytosis have highlighted their potential for clinical use to improve patient outcomes. With an estimated 1.8 million new cancer cases to be diagnosed and 600,000 cancer-related deaths to occur in the United States in 2020, the development of effective and innovative therapeutics is urgently needed more than ever (162). The up and coming field of immunotherapeutics has yielded a vast array of promising data and approaches for the treatment of different cancers. However, there remains much work to be done to continue the momentum and push forward the hopeful progress that has been established in preliminary work in this exciting avenue. This would be crucial for developing further therapies that bridge the innate and adaptive immune systems to benefit patients with cancer in a clinical setting. We hope this review inspires more studies to advance the work done and continue challenging the status quo of standard cancer care and treatment in order to improve patient outcomes.

## Author Contributions

SL, SC, and MF researched data for the article. All authors contributed to the article and approved the submitted version.

## Funding

This work was supported by the National Cancer Institute of the National Institutes of Health K99/R00 Pathway to Independence Award K99CA201075/R00CA201075 (to MF), the Damon Runyon-Dale F. Frey Award for Breakthrough Scientists DFS-22-16 (to MF), the Margaret E. Early Medical Research Trust Grant (to MF), the V Foundation for Cancer Research V Scholar Award (V2018-012) (to MF), and the startup research funding from City of Hope (to MF).

## Conflict of Interest

MF declares patent applications pertaining to stimulating TLR/BTK signaling to promote CRT in macrophages assigned to the Stanford University and equity and/or consulting with Forty Seven, Inc.

The remaining authors declare that the research was conducted in the absence of any commercial or financial relationships that could be construed as a potential conflict of interest.
